# Performance indicators of Primary Care of the *Previne Brasil* Program

**DOI:** 10.1590/1518-8345.6640.4008

**Published:** 2023-11-03

**Authors:** Tatiele Estefâni Schönholzer, Fabiana Costa Machado Zacharias, Gabriela Gonçalves Amaral, Luciana Aparecida Fabriz, Brener Santos Silva, Ione Carvalho Pinto

**Affiliations:** 1 Universidade Federal do Paraná, Curso de Medicina, Toledo, PR, Brasil.; 2 Universidade de São Paulo, Escola de Enfermagem de Ribeirão Preto, Centro Colaborador de la OPS/OMS para el Desarrollo de la Investigación en Enfermería, Ribeirão Preto, SP, Brasil.; 3 Becaria de la Coordenação de Aperfeiçoamento de Pessoal de Nível Superior (CAPES), Brasil.; 4 Universidade Estadual do Oeste do Paraná, Foz do Iguaçu, PR, Brasil.

**Keywords:** Primary Health Care, Health Evaluation, Healthcare Financing, Health Status Indicators, Pacient Care Team, Quality of Health Care, Atención Primaria de Salud, Evaluación en Salud, Financiación de la Atención de la Salud, Indicadores de Salud, Grupo de Atención al Paciente, Calidad de la Atención de Salud, Atenção Primária à Saúde, Avaliação em Saúde, Financiamento da Saúde, Indicadores de Saúde, Equipe de Saúde, Qualidade da Assistência à Saúde

## Abstract

**Objective:**

to analyze the scope of the performance indicators of the *Previne Brasil* Program of Primary Health Care.

**Method:**

an observational, descriptive study with a quantitative approach was carried out using secondary data, referring to the years 2020 and 2021, in the five Brazilian regions (North, Northeast, South, Southeast and Midwest), available in the Primary Health Care Information System. Descriptive statistics, relative frequencies and measures of central tendency and semiparametric modeling were used considering a 5% confidence interval.

**Results:**

there was evidence of evolution in the rates of performance indicators in most Brazilian regions in 2021, compared to 2020, however, the North and Midwest regions had incipient or negative rates, compared to the Southeast region. Despite the evolution in the rates of the indicators, few States managed to reach the goals established by the Ministry of Health for the strategic actions of prenatal care and women’s health; and no state achieved the goal in strategic action on chronic diseases.

**Conclusion:**

it is considered important to monitor the evolution of current indicators, envisioning their qualification so that they can evaluate primary health care and assistance, as well as guarantee the achievement of goals by ensuring funding for primary care actions.

## Abstract (es)

**Objetivo:**

analizar el alcance de los indicadores de desempeño del *Programa Previne Brasil* de Atención Primaria a la Salud.

**Método:**

para ello, se realizó un estudio observacional, descriptivo, con abordaje cuantitativo, utilizando datos secundarios, referentes a los años 2020 y 2021, en las cinco regiones brasileñas (Norte, Nordeste, Sur, Sudeste y Centro Oeste), disponibles en el Sistema de Información de la Atención Primaria de Salud. Se utilizaron estadísticas descriptivas, frecuencias relativas y medidas de tendencia central y modelación semiparamétrica considerando un intervalo de confianza del 5%.

**Resultados:**

hubo evidencia de evolución en las tasas de los indicadores de desempeño en la mayoría de las regiones brasileñas en 2021, en comparación con 2020, sin embargo, las Regiones Norte y Centro Oeste presentaron tasas incipientes o negativas, en comparación con la Región Sudeste. A pesar de la evolución en las tasas de los indicadores, pocos estados lograron alcanzar las metas establecidas por el Ministerio de Salud para las acciones estratégicas de atención prenatal y salud de la mujer, mientras que ningún estado logró la meta en la acción estratégica de enfermedades crónicas.

**Conclusión:**

se considera importante acompañar la evolución de los indicadores actuales, previendo su calificación para que puedan evaluar el seguimiento y la atención primaria en salud, así como garantizar la consecución de las metas asegurando la financiación de las acciones de atención primaria.

## Abstract (pt)

**Objetivo:**

analisar o alcance dos indicadores de desempenho do Programa Previne Brasil da Atenção Primária à Saúde.

**Método:**

realizou-se um estudo observacional, descritivo, com abordagem quantitativa, utilizando dados secundários referentes aos anos de 2020 e 2021, nas cinco regiões brasileiras (Norte, Nordeste, Sul, Sudeste e Centro-Oeste), disponíveis no Sistema de Informação da Atenção Primária à Saúde. Foram utilizadas estatística descritiva, frequências relativas e medidas de tendência central e modelagem semiparamétrica considerando o intervalo de confiança de 5%.

**Resultados:**

evidenciou-se a evolução nas taxas dos indicadores de desempenho na maioria das regiões brasileiras em 2021, comparadas com 2020, todavia as Regiões Norte e Centro-Oeste tiveram taxas incipientes ou negativas, se comparadas com a Região Sudeste. Apesar da evolução nas taxas dos indicadores, poucos estados conseguiram alcançar as metas estabelecidas pelo Ministério da Saúde para as ações estratégicas de pré-natal e saúde da mulher, enquanto nenhum estado alcançou a meta na ação estratégica de doenças crônicas.

**Conclusão:**

considera-se importante o acompanhamento da evolução dos atuais indicadores, vislumbrando a sua qualificação, para que possam avaliar a assistência e a atenção primária à saúde, bem como garantir o alcance das metas assegurando o financiamento para as ações da atenção primária.

**Highlights:**

**(1)** There was a positive evolution of performance indicators in the quarters of 2021.

**(2)** The North and Midwest regions had an incipient or negative performance.

**(3)** No state reached the goal in the strategic action of chronic diseases.

## Introduction

Primary Health Care (PHC) is characterized as one of the levels of organization of the health system, and as the organizer and coordinator of the Health Care Networks (RAS), it is configured as the preferred gateway to access the services of the Brazilian Unified Health System (SUS). The PHC has capillarization for the management of preventable diseases, reducing public spending on the aggravations, favoring the effectiveness of its attributes such as first access, continuity of care, comprehensiveness and coordination of care ^(^ 1 ^)^. 

Some difficulties are faced by the national health system, such as the political-ideological ones of universal health coverage; lack of public infrastructure; economic-financial interests related to health companies and definancing ^(^ 2 ^)^. Such problems in the Brazilian scenario, among others, are also faced by other countries that have a universal health system ^(^ 3 ^)^. 

The data generated in health services feed the Health Information Systems (SIS) and are related to the need to manage SUS, in aspects of health surveillance, production monitoring and financial transfer ^(^ 4 ^)^. In this sense, the information generated within the PHC are important tools to streamline health surveillance activities, underpinning the management of services to be provided to users and improving the quality of care ^(^ 5 ^)^. 

Until 2017, federal financial transfers for health actions for PHC were carried out through the fixed Basic Care Floor Salary (PAB) ( *per capita*) and the variable PAB (priority policies) ^(^ 6 ^)^. Thus, with the update of the National Primary Care Policy (PNAB), which revised the guidelines for the organization of Brazilian PHC, among others, it changed the composition of the financial transfer considering other resources ^(^ 7 ^)^. 

In this direction, in 2019 the *Previne Brasil* Program was established, which proposes the implementation of a new federal funding model for the cost of PHC, through the evaluation of indicators generated within the scope of the e-SUS PHC system and the Primary Care Health Information System (SISAB) ^(^ 8 ^)^. 

This program has as its principle the structuring of a financing model focused on increasing users’ access to PHC services and the link between the population and the health team. With *Previne Brasil*, the financial transfer to municipalities is now distributed based on four criteria: weighted capitation; pay for performance; incentive for strategic actions and incentive based on population criteria. Payment for performance is calculated according to results achieved indicators, monitored and evaluated, in the daily work of the family health strategy and primary care teams. The model evaluates the indicators every four months and bets on improving and increasing records in the SIS, as well as sending data to the SIS at the federal level and transparency of public spending on PHC ^(^ 8 ^)^. 

So far, the results are incipient regarding the advances and setbacks of this new financing modality. In general, the effects are worrisome on the directions of the PHC, since they tend to individualize and fragment, by suppressing the character of universalization and territorialization of the actions to be performed by the PHC teams ^(^ 6 ^,^ 9 ^)^. As for the analysis of weighted capitation or registration of the population and the analysis of the oral health indicator, they point to a significant increase in registrations in the SIS, both of teams (52 thousand) and of PHC users (50 million in the individualized and single registration) ^(^ 10 ^)^. However, there is still a lack of studies that analyze the performance criteria of health teams. 

From this perspective, it is important to measure performance indicators in order to provide results from the first years of *Previne Brasil* implementation, providing managers with parameters for strategic planning and assistance qualification. In addition, enabling the link and monitoring between the team and the user, expanding coverage and access to PHC throughout Brazil and, consequently, guaranteeing payment for the performance block. With this, the objective of this study is to analyze the scope of the performance indicators of the *Previne Brasil* Program of Primary Health Care. 

## Method

### Study design

This is an observational and descriptive study, carried out using data from the performance indicators of the *Previne Brasil* Program. The study followed the recommendations of the Strengthening the Reporting of Observational Studies in Epidemiology (STROBE). 

### Setting

Brazil is made up of 5,570 municipalities, which have a population of 213,317,639 inhabitants, distributed in 26 states, in addition to the Federal District. The country is divided into five macro-regions: North, Northeast, Midwest, Southeast and South. The macro-regions have a Municipal Human Development Index (HDI) considered medium or high, with the following values: Southeast Region (0.766), Midwest Region (0.757), South Region (0.754), North Region (0.667) and Northeast Region (0.667) ^(^ 11 ^)^. 

Regarding the proportion of health professionals *per* 1,000 inhabitants in the macro-regions, we have the following statistics for nurses and doctors, respectively: North Region (1.6 and 1.30), Northeast Region (1.76 and 1.69), Midwest Region (2.25 and 2.74), Southeast Region (2.38 and 3.15) and South Region (1.77 and 2.68) ^(^ 12 ^-^ 13 ^)^. 

### Period

Data collection took place between October 2021 and April 2022, considering that four-month data take a few weeks to be made available in the national database.

### Population

The reports of the Primary Health Care Health Information System (SISAPS) were used, which reflect the data entered by the professionals of the PHC teams during their care for the population assigned to the territory of the primary care centers, related to the four areas of strategic actions of the *Previne Brasil* Program: women’s health; prenatal; child health and chronic diseases. Each strategic action is made up of performance indicators, totaling seven. 

### Selection criteria

To be included in the study, the updating of the indicators in the information system and the availability of data download were taken into account. The indicator of inactivated poliomyelitis and pentavalent vaccine coverage, which make up the strategic action for children’s health, was not expressed in this study, given that in 2020 the achievement of 100% coverage for such vaccines was considered, due to the correction of the divergence in the calculation method in 2021 ^(^ 14 ^)^. It is observed, in this case, that the comparison analyzes would not portray the real context of the indicator in the Brazilian regions. 

### Sample definition

The universe under study was composed of the six performance indicators of *Previne Brasil*: a) Proportion of pregnant women with at least six prenatal consultations, with the first consultation up to the 20 ^th^ week of pregnancy; b) Proportion of pregnant women tested for syphilis and human immunodeficiency virus (HIV); c) Proportion of pregnant women who received dental care; d) Cytopathological test coverage; e) Percentage of hypertensive people with blood pressure checked each semester and f) Percentage of diabetics with request for glycated hemoglobin. These indicators make up three strategic actions of the *Previne Brasil* Program (prenatal care, women’s health and chronic diseases). 

### Study variables

The data were stratified taking into account the differences in the scope of each indicator between the Brazilian regions, the evolution of the records through dispersion and the results regarding the scope goals established by the Brazilian Ministry of Health, namely: a) Prenatal care, considering the indicators of “proportion of pregnant women with at least six prenatal consultations performed, the first consultation being up to the 20th week of pregnancy”, “proportion of pregnant women with syphilis and HIV tests” and “proportion of pregnant women with dental care performed”. 60% performance target; b) Women’s health, contemplating the indicator of “scope test coverage”, a performance target of 40%; c) Chronic diseases, considering the indicators of “percentage of hypertensive people with blood pressure checked each semester” and “percentage of diabetics with request for glycated hemoglobin”, performance target of 50% ^(^ 8 ^)^. 

### Instrument used for data collection

The reports generated on the SISAPS page were used, on the primary health care indicators panel, exported in comma-separated values (CSV) format and allocated in Excel spreadsheets (Microsoft Office^®^), containing the Federative Unit (FU), the four-month period (Q) and year (eg: Q1 2020, Q2 2020, Q3 2020) and the value in percentage of the rate of achievement of each indicator.

### Data collection

For data collection, the SISAPS platform available at https://sisaps.saude.gov.br/painelsaps/situacao-geral was used. For data selection, the selectable fields were used for monitoring each performance indicator agreed on in *Previne Brasil* (Period; Year; Quadrimester; Rural or urban typology; Regions of Brazil and FU). Through generated reports, the units of analysis corresponding to the 26 Brazilian states and the five macro-regions were extracted. 

### Data analysis

Data were processed and analyzed using the R Core Team 2021 program. Descriptive statistics were performed, relative frequencies and measures of central tendency were estimated, in addition to Generalized additive model modeling for location, scale and shape and categorical analysis, considering a 5% confidence interval.

### Ethical aspects

The study followed the recommendations of Resolution nº 466/2012 of the National Health Council, referring to the use of secondary data from the public and unrestricted domain of SISAPS, being unnecessary the submission of the research project for appreciation to the Research Ethics Committee involving human beings.

## Results

The results of the descriptive analysis show, in Table 1, that the indicator with the highest coverage (46.2%) in Brazil was the proportion of pregnant women who underwent syphilis and HIV tests, with a minimum of 18% and a maximum of 72%. On the other hand, the indicator with the lowest coverage (6.5%) was the percentage of hypertensive people with blood pressure measured, with a minimum of 0% and a maximum of 20%. 

**Table 1 -:** Performance indicators of Primary Health Care of the *Previne Brasil* Program, according to measures of central tendency, standard deviation and quartiles, year 2021. Brazil, 2022

Indicators	Mean	SD*	Mín ^†^	Q‡0.25	Q ^‡^0.5	Q ^‡^0.75	Max ^§^
Proportion of pregnant women with at least 6 (six) prenatal consultations, from the 1 ^st^ to the 20 ^th^ week of pregnancy.	34.7	10.9	6.0	28.0	34.0	42.7	56.0
Proportion of pregnant women tested for syphilis and HIV ^||.^	46.2	13.2	18.0	38.0	44.5	57.0	72.0
Proportion of pregnant women with dental care performed.	25.7	12.6	6.0	17.0	23.0	32.0	68.0
Pap smear coverage.	14.2	4.0	4.0	12.0	15.0	17.0	24.0
Percentage of hypertensive people with blood pressure measured each semester.	6.5	4.0	0.0	4.0	6.0	8.7	20.0
Percentage of diabetics requesting glycated hemoglobin	12.6	8.4	2.0	6.2	11.0	17.0	49.0

Source of secondary data: SISAPS, 2022. ^*^SD = standard deviation; ^†^Min = Minimum; ^‡^Q = Quartile; ^§^Max = Maximum; ^||^HIV = Human immunodeficiency virus

**Figure 1 -:**
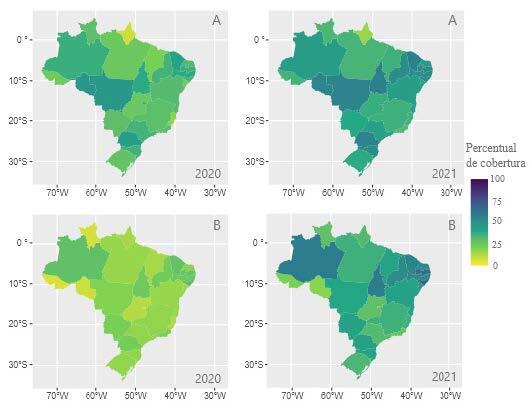
Performance indicators of the *Previne Brasil* Program, referring to the strategic action of prenatal care in the third quarter of 2020 and 2021. Brazil, 2022

**Figure 2 -:**
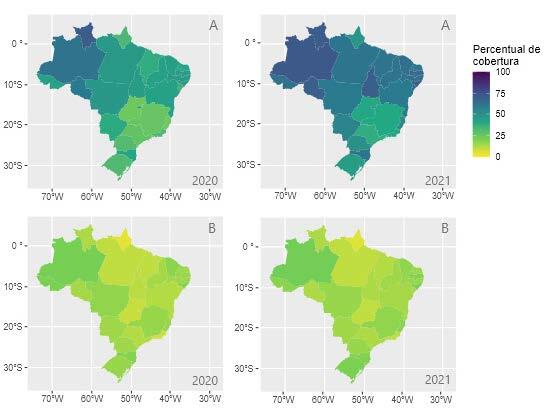
Performance indicators of the *Previne Brasil* Program, referring to the strategic action on prenatal care and women’s health in the third quarter of 2020 and 2021. Brazil, 2022

**Figure 3 -:**
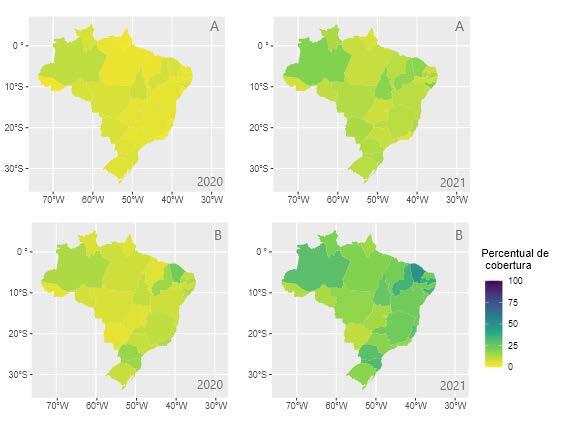
Performance indicators of the *Previne Brasil* Program, referring to the strategic action of chronic diseases in the third quarter of 2020 and 2021. Brazil, 2022

In Figures 1, 2 and 3 below, there are the results of the evolution in the rates of performance indicators in PHC. In this regard, the increase in the performance of the indicators in the Brazilian regions is visible, when comparing the dispersion between 2020 and 2021. When performing the descriptive analysis and comparing with the goals established by the Ministry of Health, it is evident that, despite the increase in records, many states were unable to reach the recommended goals. 

In Figure 1A, despite an increase in the dispersion of the indicator “percentage of pregnant women who received prenatal care” in the third quarter of 2021 compared to 2020, only four states reached the 60% target (Ceará, Mato Grosso, Tocantins and Paraná). In Figure 1B, there is a significant increase in dispersion regarding the number of dental appointments in pregnant women, however, two states managed to reach the 60% target established by the Ministry of Health (Alagoas and Paraíba). The States of Amazonas and Tocantins approached the established target, reaching 58%. 

Regarding the dispersion of the indicator, in the third quarter of 2021, Figure 2A, it is noted that, despite the increase in HIV and syphilis tests during pregnancy, seven states failed to reach the 60% performance target (Espírito Santo, Amapá, Paraná, Rio Grande do Sul, Rio de Janeiro, Goiás, Minas Gerais and São Paulo). In Figure 2B, it is observed that no State managed to reach the goal of 40% of Pap smears being performed in PHC. 

Caption: A – Proportion of pregnant women with at least 6 (six) prenatal consultations, with the 1 ^st^ up to the 20 ^th^ week of pregnancy). B - Proportion of pregnant women with dental care performed. The colors symbolize the percentage of coverage/achievement of the targets ranging from 0% (light yellow) to 100% (dark blue); *Percentual de cobertura* = Coverage percentage

Caption: A – Proportion of pregnant women tested for syphilis and HIV; B - Coverage of cytopathological examination. The colors symbolize the percentage of coverage/achievement of the targets ranging from 0% (light yellow) to 100% (dark blue); *Percentual de cobertura* = Coverage percentage

In Figure 3A and Figure 3B, a slight increase in the dispersion of records is observed in some regions, however, no State managed to reach the 50% target recommended for the request of glycated hemoglobin for diabetics and blood pressure measurement for hypertensives. It should be noted that Ceará was the state that most approached the target, reaching 49%. 

Caption: A – Percentage of hypertensive people with blood pressure measured each semester. B - Percentage of diabetics requesting glycated hemoglobin. The colors symbolize the percentage of coverage/achievement of the targets ranging from 0% (light yellow) to 100% (dark blue); *Percentual de cobertura* = Coverage percentage

Table 2 shows the results of the analysis of the six performance indicators of the *Previne Brasil* Program, the strategic action on prenatal care, women’s health and chronic diseases, agreed for the years 2020 and 2021. 

The results of the indicator “Proportion of pregnant women with at least 6 (six) prenatal consultations performed, from the 1 ^st^ to the 20 ^th^ week of pregnancy”, described in Table 2, showed a relative increase of 66.5% in the average in 2021 in relation to the year 2020. A total of 9.7% and in the South region of 46.9% in relation to the Southeast region. 

With regard to the indicator “Proportion of pregnant women who underwent tests for syphilis and HIV” ( Table 2), in the year 2021, a relative increase in the average of 86% was observed compared to the year 2020 with the Southeast region. 

**Table 2 -:** Categorical analysis of the performance indicators of Primary Health Care in the five Brazilian regions, quarters and years 2020 and 2021. Brazil, 2022

Proportion of pregnant women with at least six prenatal consultations, from the 1 ^st^ to the 20 ^th^ week of pregnancy							
Categories	Estimate	Standard error	Valor t	Pr(>|t|)*	Interval-RI ^†^		
					Medium	Lower	Upper
Categorical mean	-1.24	0.03	-44.27	0.0000*			
Quarter 2	0.04	0.02	1.88	0.06	1.04	1.00	1.09
Quarter 3	0.22	0.02	10.52	0.0000*	1.25	1.20	1.30
Year 2021	0.51	0.02	29.41	0.0000*	1.67	1.61	1.72
Midwest	0.34	0.03	10.59	0.0000*	1.40	1.32	1.50
North East	0.36	0.03	12.98	0.0000*	1.43	1.35	1.51
North	0.09	0.03	3.18	0.0019*	1.10	1.04	1.16
South	0.38	0.03	11.23	0.0000*	1.47	1.37	1.57
Proportion of pregnant women tested for syphilis and HIV ^‡^							
Categories	Estimate	Standard error	Valor t	Pr(>|t|)*	Interval-RI ^†^		
					Medium	Lower	Upper
Categorical mean	-1.19	0.03	-35.51	0.0000*			
Quarter 2	0.09	0.03	3.76	0.0003*	1.10	1.05	1.15
Quarter 3	0.26	0.03	10.33	0.0000*	1.30	1.23	1.36
Year 2021	0.62	0.02	30.31	0.0000*	1.86	1.79	1.94
Midwest	0.48	0.04	12.46	0.0000*	1.62	1.50	1.74
North East	0.73	0.03	22.36	0.0000*	2.08	1.95	2.21
North	0.95	0.03	27.65	0.0000*	2.58	2.41	2.76
South	0.34	0.04	8.32	0.0000*	1.41	1.30	1.53
Proportion of pregnant women with dental care performed							
Categories	Estimate	Standard error	Valor t	Pr(>|t|)*	Interval-RI ^†^		
					Medium	Lower	Upper
Categorical mean	-2.07	0.05	-42.09	0.0000*			
Quarter 2	0.38	0.04	10.72	0.0000*	1.47	1.37	1.58
Quarter 3	0.74	0.05	15.17	0.0000*	2.09	1.90	2.30
Year 2021	0.81	0.03	31.25	0.0000*	2.26	2.14	2.37
Midwest	-0.16	0.02	-9.65	0.0000*	0.85	0.82	0.88
North East	0.56	0.02	34.63	0.0000*	1.76	1.70	1.81
North	-0.27	0.02	-13.73	0.0000*	0.77	0.74	0.80
South	0.13	0.02	6.69	0.0000*	1.14	1.09	1.18
Pap smear coverage							
Categories	Estimate	Standard error	Valor t	Pr(>|t|)*	Interval-RI ^†^		
					Medium	Lower	Upper
Categorical mean	-1.87	0.02	-98.01	0.0000*			
Quarter 2	-0.03	0.01	-2.33	0.0214*	0.97	0.94	0.99
Quarter 3	0.01	0.01	0.53	0.60	1.01	0.98	1.04
Year 2021	0.00	0.01	-0.32	0.75	1.00	0.97	1.02
Midwest	-0.08	0.02	-3.68	0.0003*	0.92	0.88	0.96
North East	0.14	0.02	7.48	0.0000*	1.15	1.11	1.20
North	-0.03	0.02	-1.39	0.17	0.97	0.93	1.01
South	0.31	0.02	13.33	0.0000*	1.36	1.30	1.42
Percentage of hypertensive people with blood pressure measured each semester							
Categories	Estimate	Standard error	Valor t	Pr(>|t|)*	Interval-RI ^†^		
					Medium	Lower	Upper
Categorical mean	-3.80	0.05	-74.92	0.0000*			
Quarter 2	0.32	0.03	11.74	0.0000*	1.37	1.30	1.45
Quarter 3	0.61	0.03	21.56	0.0000*	1.84	1.74	1.94
Year 2021	0.86	0.04	22.82	0.0000*	2.37	2.20	2.56
Midwest	0.19	0.04	5.41	0.0000*	1.21	1.13	1.29
North East	0.32	0.03	11.50	0.0000*	1.38	1.31	1.46
North	0.24	0.03	8.62	0.0000*	1.27	1.20	1.34
South	0.34	0.02	13.73	0.0000*	1.41	1.34	1.48
Percentage of diabetics requesting glycated hemoglobin							
Categories	Estimate	Standard error	Valor t	Pr(>|t|)*	Interval-RI ^†^		
					Medium	Lower	Upper
Categorical mean	-2.94	0.05	-54.42	0.0000*			
Quarter 2	0.39	0.03	14.44	0.0000*	1.47	1.40	1.55
Quarter 3	0.72	0.03	27.58	0.0000*	2.06	1.96	2.17
Year 2021	0.96	0.04	24.42	0.0000*	2.61	2.42	2.82
Midwest	-0.63	0.04	-16.81	0.0000*	0.53	0.50	0.57
North East	0.32	0.04	8.27	0.0000*	1.38	1.28	1.49
North	-0.22	0.04	-6.10	0.0000*	0.80	0.75	0.86
South	0.23	0.04	5.48	0.0000*	1.26	1.16	1.37

*Pr(>|t|) = P-value associated with the value in the value column t; ^†^RI = Relative increase

The results of the indicator “Proportion of pregnant women with dental care performed” ( Table 2) show that in the year 2021 there was a relative increase in the average of 125.6% in relation to the year 2020. When stratifying the regions, it appears that the Northeast region had a relative increase in the average of 75.7% and the South region of 13.5%, in relation to the Southeast region. On the other hand, there was a relative decrease in the average in the North and Midwest regions, of 23.5% and 14.9%, respectively, when compared to the Southeast region. 

Concerning the results of the indicator “Cyptopathology test coverage” ( Table 2), comparing the years 2020 and 2021, there was no significant difference; however, comparing the indicators between regions, it is noted that in the Northeast and South regions there was a relative increase in the average, of 15.29% and 36.16%, respectively, compared to the Southeast region. However, in the Midwest region, there is a reduction of 8.13% in the average, when compared to the Southeast region. 

In the results of the indicator “Percentage of hypertensive people with blood pressure measured in each semester”, in the year 2021 ( Table 2), there was a relative increase in the average of 161.3% in relation to the year 2020, in Brazil. Following this progress, there is a relative increase in the average in the Northeast and South regions of 75.7% and 26.3%, respectively, compared to the Southeast region. However, in the North and Central-West regions there was a relative reduction on average of 19.8% and 46.6%, if compared to the Southeast region. 

As for the “Percentage of diabetics requesting glycated hemoglobin”, in the year 2021 ( Table 2) there was a relative increase in the average of 133.4%, compared to the year 2020, throughout the Brazilian territory. In this indicator, all regions followed the growth, thus, a relative increase of the average 20.9% in the Center-West region, 38% in the Northeast region, 26.8% in the North region and, in the South region, 40.5%, if compared to the Southeast region. 

## Discussion

This study shows, with unprecedented evidence, the evolution of the records of the first PHC performance indicators of the *Previne Brasil* Program, agreed for the years 2020 and 2021. The results for the strategic actions of prenatal care and women’s health show a significant increase in records in the year 2021. 

Corroborating the findings of this study, a survey carried out with data from the three cycles of the Access and Quality Improvement Program of PHC (PMAQ) shows that the two regions that had less access to dental consultations during pregnancy were the Midwest and North regions ^(^ 15 ^)^. Other studies showed that there was a decrease in prenatal quality indicators, with a reduction in the number of consultations during pregnancy, from 2012 to 2018 ^(^ 16 ^)^, and low dental follow-up (24.5% of pregnant women underwent some follow-up in the studied setting) ^(^ 17 ^)^. In addition, 24% of Brazilian municipalities had reasonable prenatal care, with weaknesses in terms of structure, operational aspects, access, promotion, prevention and follow-up of care ^(^ 18 ^)^. 

A study shows that factors such as welcoming and opening hours that meet the needs of pregnant women represented, respectively, 74% and 85.9% of the use of oral health services during prenatal care ^(^ 15 ^)^. Low adherence to dental appointments during pregnancy may be related to beliefs that it is harmful to fetal development and to lack of information on the part of users, in addition to insecurity on the part of professionals. Likewise, barriers to accessing the health service influence low adherence ^(^ 19 ^)^. 

Concerning maternal and child health, studies that correlated the MHDI with this binomial found an association with rates of maternal near death ^(^ 20 ^)^ and infant death in the first year of life ^(^ 21 ^)^_._ Comparing the regions of Brazil, infant mortality rates were also associated with the MHDI, as the northern region (Amapá, Roraima and Amazonas) and the Midwest region (Mato Grosso) had an infant death rate above the national average ^(^ 22 ^)^. 

The results of the strategic action indicators for chronic conditions showed a significant increase in records considering the year 2021. In contrast to this increase, the North and Midwest regions had a decrease in the percentage of hypertensive people with blood pressure measured. However, they positively accompanied the increase in records regarding the request for glycated hemoglobin for people with diabetes.

Regarding the follow-up and relevance of requesting HbA1c tests for people with diabetes, studies showed that the request was considered adequate, however, 30% of users did not receive the test request ^(^ 23 ^)^ and, in another study, 30% of users reported that they did not do the test ^(^ 24 ^)^. Also in this sense, alterations were observed in more than half of the tests of users with diabetes, indicating the lack of adequate control of the pathology. It is evident that other important tests for monitoring users with diabetes, such as the eye fundus exam and foot exam, were neglected mainly in the North, Northeast and Midwest regions ^(^ 23 ^)^. 

PHC has a fundamental role in the surveillance process of chronic diseases, with health promotion and prevention actions, continuous monitoring and coordination of care in the health care network ^(^ 25 ^)^. In 2017, 90% of the PHC centers had specific protocols for users with diabetes. However, when analyzing the PHC attributes, the longitudinality and coordination of care were poorly evaluated, impacting the continuity of care and access to specialized care ^(^ 26 ^)^. Another worrying factor is the number of cases of underreported diabetic users in the country, around 40%, being more expressive in the North region, with around 73% ^(^ 23 ^)^. 

A study carried out in two municipalities in the Northeast region, on the assessment of blood pressure control of users, showed that more than half of the hypertensive patients registered in the *Hiperdia* Program were not monitored by the PHC service and, of those monitored, more than 60% did not have their blood pressure controlled ^(^ 27 ^)^. Another study, in Mexico, which evaluated the evolution of kidney disease in hypertensive users followed by the PHC, found that the glomerular filtration rate decreased by 5.8 ml/min/year in the first seven years ^(^ 28 ^)^. Therefore, the measurement of blood pressure and the proper management of hypertensive users are of paramount importance for the prevention, diagnosis and monitoring of blood pressure values, as well as the aggravation and involvement of target organs ^(^ 29 ^)^. 

It should be noted that the implementation of the *Previne Brasil* Program and the evolution of performance indicator records may have been impacted by the coronavirus disease (COVID-19) pandemic. It is understood that social isolation and fear affected the population, reduced the contact of users with health services, as well as the service protocols of health centers to assist users, as they were initially suspended and later modified (reduced or adapted) ^(^ 30 ^)^. Still in this sense, the Brazilian states responded differently to the COVID-19 epidemic, in relation to the number of deaths. A study shows that the states of Amazonas, Mato Grosso and Rondônia had the highest number of deaths *per* 100,000 inhabitants, which may be related to the difficulties faced, in this period, in health care processes ^(^ 31 ^)^. 

In this sense, each performance indicator has specificities in its calculation, both in the registration and in the individual consultations carried out by PHC professionals, with the registration of the International Classification of Diseases (ICD10) or International Classification of Primary Care (CIAP2) in the PHC information system, adopted in Brazil ^(^ 32 ^)^. Thus, the non-registration by the professionals or the registration in a non-recommended way, due to the lack of training ^(^ 33 ^)^, may have had an impact on the calculation and, consequently, on the evolution of performance indicator rates. This fact may explain, in this study, the low achievement of goals, mainly referring to indicators of chronic diseases, as these rates differ from those published by the PMAQ in 2018 ^(^ 34 ^)^. 

With regard to the assessment and monitoring of PHC in Brazil, some initiatives were carried out previously, such as the proposal to create a portfolio of PHC services; the use of Primary Care Assessment instruments; the Interfederative Pact of Indicators (SISPACTO); the PMAQ; the Primary Care Evaluation and Monitoring Program, in the state of Paraná – Quali AB ^(^ 35 ^-^ 36 ^)^. 

Regarding the *Previne Brasil* Program, the indicators that include the payment-for-performance incentive were chosen via agreement in the Tripartite Intermanagement Commission (TIC), which listed the areas of priority actions based on the Brazilian epidemiological and clinical relevance. Through planning, it was intended to implement 21 indicators, contemplating other strategic actions, by the year 2022 ^(^ 37 ^)^, a fact that did not occur due to the non-achievement of the goals, established by the Ministry of Health, for the Brazilian states. By July 2022, only seven Brazilian municipalities had reached 100% of the Final Synthetic Indicator of coverage of the seven indicators, these being small municipalities (< 25,000 inhabitants). 

In this sense, there is concern about the scope of indicators to translate the reality of the population and PHC services, in their different contexts and scenarios. There is a risk of regression regarding the structural model of the Family Health Strategy ^(^ 38 ^)^ and apprehension about funding ^(^ 39 ^)^. 

Thus, a study analyzed the change in financing of *Previne Brasil* with the predecessor PAB model. It showed a strong correlation between the amount received from both programs, however, due to the redistribution between the payment pillars, some municipalities earn more (depending on the type of municipality and with greater vulnerability) and others less (cities with the same population size and with lower *per capita* income). With regard specifically to the pillar of performance indicators, until 2022 there was no decrease in resources allocated to municipalities, however, the payment in this period still did not consider the actual scope of the indicators ^(^ 40 ^)^, due to the difficulty of implementing the program during the pandemic. 

According to the Lancet Global Health commission, the financing of PHC in the world becomes a global challenge due to its heterogeneity in its organization and different understanding by each country, and often, the forms of arrangement by which PHC is financed are not clear. Thus, considering that resource allocation should be centered on health needs, combined mechanisms become the best financing option. In this sense, fundraising is a good resource to link the population to the service and the performance analysis enables adaptation to other objectives to be achieved by the health system ^(^ 41 ^)^, these being incorporated into *Previne Brasil*. 

For PHC performance indicators, whether for evaluation, funding or both, studies show heterogeneity in their quantity ^(^ 42 ^-^ 43 ^)^_,_ reinforcing the importance of adapting the indicators to local needs (assistance and administrative). However, for Brazil, it is expected the expansion of indicators for better coverage of services performed in PHC (individual or collective), as well as records by other professionals who work in health teams, allowing a global view of assistance to users. 

From the point of view of change management, it is up to local managers and health professionals to assess the progress of records, the long-term implication of the indicators in the population’s access to health and the difficulties encountered by the teams in this process. In this sense, nursing plays an important role in monitoring the records in the PHC information system, in achieving the goals of *Previne Brasil*, in the territory where it operates, in the management of services and in individual and collective care. 

This study contributes, in an unprecedented way, with the evaluation of the scope of performance indicators, in the first years of implementation, indicating possible difficulties in some Brazilian regions, which serves as a warning for the planning of strategic actions and in the evaluation of the new financing model of the Brazilian PHC. As a limitation, the absence of analysis of the vaccination coverage indicator was considered, which made it difficult to analyze the general panorama of the first agreed block, with seven indicators, of the *Previne Brasil* Program. 

## Conclusion

This study presented the evolution of the performance indicator rates of the APS financing program, *Previne Brasil*. It was found that there was a significant evolution between the three quarters, by region of Brazil, in the year 2021. In the strategic prenatal action, the region with the lowest rate in the three indicators was the North Region. In the strategic action of women’s health, in the cytopathological coverage indicator, the Midwest Region had the lowest rate; and in the strategic action of chronic diseases, in the indicator of percentage of hypertensive people with blood pressure checked, the regions with the lowest rates were the North and Midwest. From the point of view of reaching the goals established by the Ministry of Health for each performance indicator, only a few states managed to reach such goals in the strategic actions of prenatal care and women’s health and no Brazilian state reached the goal in the strategic action of chronic diseases. 

As a future perspective, it is worth analyzing how the evolution of the implemented performance indicators, included in this study, will behave after reaching the goals established in the *Previne Brasil* Program, and with the insertion of the other indicators. Other aspects concern the qualification of the indicators in the assessment of the PHC and in the confrontation by the management and health team regarding the obstacles concerning the goals not achieved, and which were evidenced in the studies related to the PMAQ indicators. 
